# Role of cystatin C in urogenital malignancy

**DOI:** 10.3389/fendo.2022.1082871

**Published:** 2022-12-14

**Authors:** Li Ding, Zijie Liu, Junqi Wang

**Affiliations:** ^1^ Department of Urology, the Affiliated Hospital of Xuzhou Medical University, Xuzhou, Jiangsu, China; ^2^ Department of Urology, Wuxi No.2 People’s Hospital, Nanjing Medical University, Wuxi, Jiangsu, China

**Keywords:** urogenital malignancy, cystatin C, proteinase inhibitor, renal function, survival prediction, tumor progression

## Abstract

Urogenital malignancy accounts for one of the major causes of cancer-related deaths globally. Numerous studies have investigated novel molecular markers in the blood circulation, tumor tissue, or urine in order to assist in the clinical identification of tumors at early stages, predict the response of therapeutic strategies, and give accurate prognosis assessment. As an endogenous inhibitor of lysosomal cysteine proteinases, cystatin C plays an integral role in diverse processes. A substantial number of studies have indicated that it may be such a potential promising biomarker. Therefore, this review was intended to provide a detailed overview of the role of cystatin C in urogenital malignancy.

## Introduction

Urogenital malignancies are a spectrum of fatal cancers that affect the urinary and/or reproductive organs (1, 2). Prostate cancer, as the most prevalent cancer in men, accounts for approximately 27% of all new cancer cases and is also the second most common cause of cancer-related deaths. Renal cell carcinoma (RCC) and bladder cancer are both extremely common malignant tumors of the urinary system in both genders (1). Early detection of tumors may lead to better treatment response and a more favorable survival prognosis. Meanwhile, prompt and accurate diagnosis may reduce hospitalization costs and make treatment less difficult (3, 4). Therefore, the search for novel biomarkers to assist clinical decision making in urogenital malignancies has become a hot research topic.

Cystatin C is stably expressed in all nucleated cells and is involved in various physiological or pathological conditions (5). It is often used clinically to assess renal function because of its relatively small molecular weight and ease of detection, rendering it an ideal marker of the glomerular filtration rate (GFR) (6). Whereas, there is growing evidence that cystatin C is involved in a variety of immune responses. Under pathological conditions, if not properly controlled, it may eventually lead to the development and progression of malignant tumors (5, 7, 8).

## Structure and function

Cystatin is a natural cysteine protease inhibitor that is widely present in the human body (9). The cystatin superfamily is divided into three subtypes based on their amino acid sequence and three-dimensional molecular structure. Type I cystatins, including stefins A and B, are non-glycosylated proteins that do not have disulfide bonds and are found primarily in cells, but can also be detected in body fluids. Type II cystatins include cystatin C, D, E/M, F, G, S, SN, and SA (5). Type III cystatins are kininogenes that can be detected in plasma and other body fluids (5, 10, 11).

**Figure 1:**
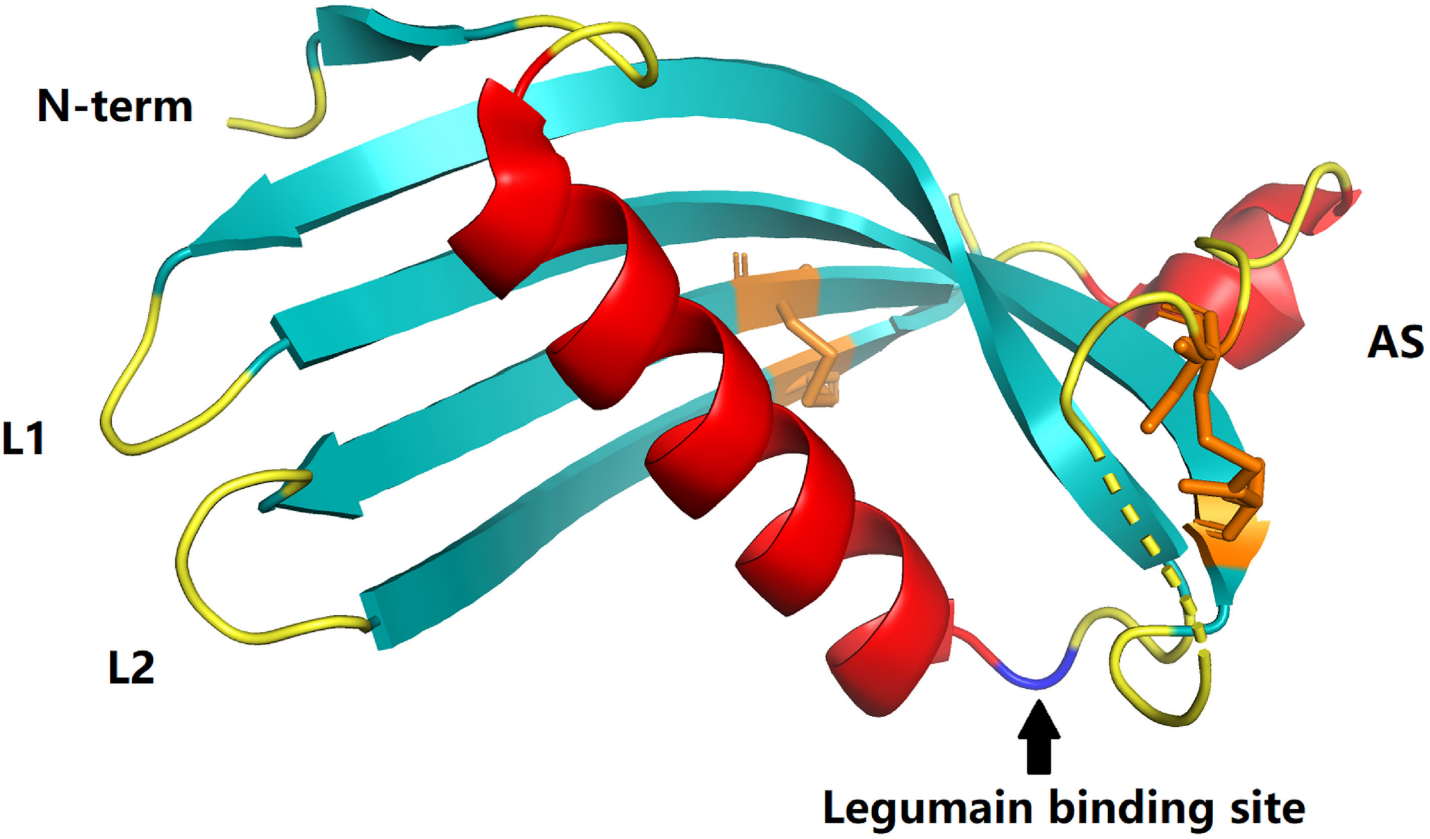
Crystal structures of cystatins C (PDB:3GAX). The papain-binding epitope is formed by the N-terminus, loop L1, and loop L2. The AS is an irregular appending structure at a ‘back side’ loop system harboring a potential legumain-binding site (blue). Disulfide bonds in cystatin C are shown in orange.

Cystatin C, as the most potent known inhibitor of cysteine peptidases (5), strongly suppresses the activity of papain-like cysteine proteases and legumain (9) (**Figure 1**). It is a small (13-kDa) alkaline secreted protein encoded by the CST3 housekeeping gene located on chromosome 20 (20pl1.21). The human mature, active cystatin C, a single non-glycosylated polypeptide chain (molecular weight 13.4 kDa), consists of 120 amino acid residues with two intracellular disulfide bonds at the C-terminus (**Figure 1**) (5, 9, 12–14). Cystatin C can be detected in most tissues and organs of the human body (9, 12, 13), but varies greatly in different body fluids, with the highest levels in semen, while it is almost undetectable in urine (9, 12, 15). Cystatin C concentrations in serum are about 0.6-1.2 mg/L in healthy adults, with a half-life of approximately two hours (9, 12). Under physiological conditions, cystatin C levels remain stable. Gender, age, altered hormone levels, alcohol intake, etc. do not cause pronounced fluctuations in their levels (6, 13, 16, 17). Kidney is the main catabolic site. Cystatin C is almost completely freely filtered by the glomerulus and is then enzymatically degraded after complete reabsorption in the proximal tubule. These properties make it meet most of the criteria for ideal markers of GFR (14, 18, 19). Therefore, it has long been considered as an indicator to evaluate renal function (6, 16). Even more importantly, accumulated evidence indicates that cystatin C may be more accurate than traditional serum creatinine in renal function evaluation (20–28).

**Figure 2:**
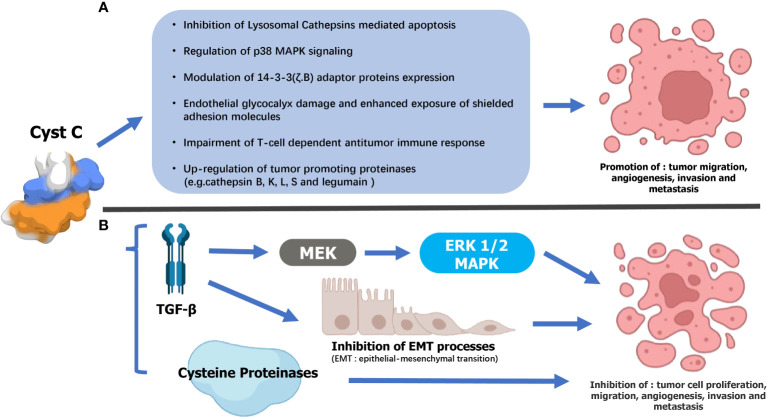
Supposed mechanisms underlying the dual opposing effects of Cystatin C (Cyst C) on urogenital malignancy progression. **(A)** Tumor-suppressing mechanisms 1) Cathepsin inhibition-dependent: Cyst C may thwart urogenital malignancy progression by inhibiting extracellular cysteine cathepsin activity and, consequently, their contribution to tumor cell migration, invasion, angiogenesis, and metastasis. 2) TGF-β interaction: Cyst C inhibited TGF-β binding to its type II cell surface receptor as well as TGF-β stimulation of initiating metastatic events such as epithelial-to-mesenchymal transition (EMT). 3)Cyst C may also mediate tumor cell invasion by regulating the MAPK/ERK cascade. **(B)** Cyst C may promote the malignant progression of urogenital malignancy by 1) inhibiting Lysosomal Cathepsins-mediated apoptosis. 2)Regulation of p38 MAPK signaling. 3)Modulation of 14-3-3 (ζ.B) adaptor proteins expression. 4) Damage to the endothelial glycocalyx and increased exposure of shielded adhesion molecules. 5) Impaired antitumor immune response mediated by T cells. 6) Increased expression of tumor-promoting proteinases (for example, cathelipsin B, K, L, S, and legumain).

In addition to the aforementioned regulation of intracellular and extracellular lysosomal cysteine proteins, cystatin C also exerts a variety of important functions (5, 14), including cell proliferation (29, 30), cell differentiation (30–34), cell migration (35, 36), immune regulation (31, 37), neuroprotection (38), resistance to microbial and viral infections (33), etc. Non-physiological fluctuations of cystatin C levels can be found in tumor tissues and body fluid of patients, including RCC (39–42), bladder cancer (43, 44), prostate cancer (45, 46), etc. Though the exact role of cystatin C in cancer still needs to be well characterized, some possible mechanisms are reported in previous studies (5, 14) (**Figure 2**).

## Cystatin C and renal cell carcinoma

### Circulating cystatin C expression in renal cell carcinoma

Preoperative and postoperative serum cystatin C levels may offer potential predictive value for postoperative renal function impairment in RCC patients (40, 47, 48). Studies by Duan et al. (47) and Zheng et al. (48) both concluded that serum cystatin C level was correlated with early postoperative renal impairment but was of insufficient value when predicting renal function. In the study by Wenzel et al. (40), using logistic regression and linear regression to analyse 195 patients who had undergone nephrectomy, they found that elevated preoperative cystatin C (odds ratio: 18.3, P < 0.01) and partial operation (odds ratio: 13.5, P < 0.01) were independent predictors of lower estimated GFR (eGFR) < 60 ml/min/1.73m^2^ at follow-up, whereas serum creatinine was not. They found that when postoperative cystatin C levels were elevated in the range of 0.9-1.0 mg/l, postoperative creatinine levels remained stable at around 1.3 mg/dl, while cystatin C and creatinine levels remained highly correlated when postoperative cystatin C levels were below 0.9 mg/l or above 1.0 mg/l. The authors referred to the postoperative plateau period of 1.2-1.3 mg/dl of creatinine as the “creatinine blind area” during which fluctuations in cystatin C levels might better predict renal impairment. Within the “creatinine blind area”, postoperative renal impairment is undetectable in up to one-third of patients, yet within this range, significant changes in serum cystatin C levels can be observed in these patients. The finding may change the traditional definition of creatinine-based acute kidney injury (AKI).

Three studies evaluated the predictive value of serum cystatin C level on RCC patients’ prognosis (39, 41, 49). As shown in **Table 2**, serum cystatin C levels did not change significantly in metastatic patients compared to patients with localized RCC. The cut-off values of serum cystatin C in three papers were all in the range of around 1.1 mg/l, which was broadly consistent with the levels of patients with other cancer types (8, 14). All three studies suggested that there is a significant correlation between elevated serum cystatin C levels and poorer prognosis. Guo et al. (49) and Zhao et al. (41) focused their studies on nephrectomy patients. Both studies had similar sample sizes of more than 300 cases, and by multivariate Cox regression analysis, both studies showed that serum cystatin C levels were an independent predictor of prognosis. In the study by Guo et al. (49), when predicting overall survival (OS), the hazard ratio (HR) for high levels of cystatin C was 1.59 (P = 0.012). When disease-free survival was predicted, the HR for high levels of cystatin C was 3.50 (P = 0.013). And in the study by Zhao et al. (41), when predicting OS, the HR for high levels of cystatin C was 10.51 (P = 0.001). When cancer-specific survival (CSS) was predicted, the HR for high levels of cystatin C was 4.944 (P = 0.048). In a phase II clinical study in patients with metastatic renal cell carcinoma (mRCC), Bodnar et al. (39) evaluated the impact of serum cystatin C levels relative to other GFR markers on treatment outcomes during everolimus treatment. They found a significant connection between cystatin C level and GFR indicators in 56 subjects who underwent analysis (R Spearman from 0.69 to 1.00; P = 0.0001). The result showed that elevated cystatin C level [HR = 2.85, 95% confidence interval (CI) 1.34-6.05, P=0.0065] was an independent predictor of worse treatment outcome with everolimus. Multivariate analysis showed that patients with elevated pre-treatment cystatin C levels had poorer OS (HR = 2.60, 95% CI 1.03-2.60, P = 0.0428). The results suggest that, compared to other GFR markers (such as the Modification of Diet in Renal Disease equation, the Cockcroft-Gault equation, etc.), elevated serum cystatin C levels are better predictors of mRCC patients and had better predictive significance.

### Cystatin C expression in renal cell carcinoma tissue

In 1995, Jacobsson et al. (50) investigated the presence of transthyretin mRNA and cystatin C mRNA in 10 normal kidney specimens and 32 renal cell carcinoma lesions using Northern blot analysis, and immunohistochemistry was performed on some of these specimens. They found very low amounts of CST3 mRNA in the samples and ruled out the possibility of using cystatin C as the specific tumor marker for RCC. Guo et al. (42) used immunohistochemistry and Western blotting to determine the degree of cystatin C expression in 253 clear cell renal cell carcinoma (ccRCC) tissues. The researchers looked at the correlation between cystatin C expression levels and the clinicopathological features of ccRCC tumors. Their findings revealed that cystatin C expression levels in ccRCC tissues were lower than in surrounding non-tumor tissues (P < 0.001). Patients with low cystatin C expression in tumor tissues had a longer OS than those with elevated cystatin C expression. Furthermore, in the 786-O RCC cell line, knocking down cystatin C hindered cell proliferation, caused G0/G1 arrest, repressed cell invasion, decreased phosphorylation of ERK1/2 and STAT3, and increased phosphorylated JNK expression. These findings showed that cystatin C in tissues might be an excellent prognostic indicator in ccRCC.

### Urine cystatin C expression in renal cell carcinoma

Lane et al. (51) attempted to identify valid markers that could predict early AKI after partial nephrectomy by measuring fluctuations in the levels of multiple urinary biomarkers after partial nephrectomy. However, the results showed that multiple urinary biomarkers, including cystatin C, showed only slight and transient fluctuations after partial nephrectomy. Urinary cystatin C levels did not correlate significantly with long-term renal function changes. There was no significant correlation between cystatin C and parameters such as time to surgery and time to ischemia. Therefore, cystatin C is not a meaningful factor for the early prediction of AKI after partial nephrectomy.

## Cystatin C in prostate cancer

### Circulating cystatin C expression in prostate cancer

Südfeld et al. (23) analyzed the effect of hydroxyethyl starch on cystatin C-derived eGFR by including 179 prostate cancer patients who underwent radical prostatectomy under general anesthesia and were administered hydroxyethyl starch during the perioperative period. The results showed that perioperative application of 1000 ml of 6% hydroxyethyl starch did not impair renal function in the early postoperative period when the patient’s baseline renal function was not significantly abnormal. Yordanova et al. (45) evaluated renal function by measuring creatinine, GFR, and cystatin C in 55 CRPC patients treated with at least three cycles of [^177^Lu] Lu-PSMA-617 radioligand and showed that cystatin C was one of the most reliable predictive markers of nephrotoxicity. At baseline, serum cystatin C was elevated in only 14 patients. However, cystatin C was elevated in 32 patients (58%) after treatment. Yang et al. (24), by comparing the preoperative cystatin C levels in three patient groups (benign prostatic hyperplasia, intraepithelial neoplasia, and confirmed prostate cancer), did not find any statistical difference between the three groups (P > 0.05). They also found that age and serum creatinine influenced the changes in serum cystatin C levels in PCa patients to some extent (both P < 0.001). Therefore, they concluded that preoperative cystatin C levels cannot be used for the early diagnosis of prostate cancer but can assist in predicting renal function in patients with prostate cancer.

Tumminello et al. (52) attempted to assess the clinical significance of serum cystatin C in prostate cancer patients without distant metastases or with bone metastases only. Circulating cystatin C levels were higher in prostate cancer patients than in healthy blood donors (P = 0.0001) and in patients with BPH (P = 0.0078). Therefore, serum cystatin C may be an effective tumor marker for differentiating prostate cancer from benign prostate lesions. However, several indicators, including the number of bone metastases and cystatin C levels, did not reveal any further relationship with the progression of cancer. It is worth noting that they also found significantly elevated cystatin C levels in prostate cancer patients treated with zoledronic acid, implying that cystatin C may be a potential marker for monitoring treatment response after receiving bisphosphonate-type drugs in prostate cancer patients who develop bone destruction. To find novel prostate cancer biomarkers, Larkin et al. (53) applied enhanced proteomic profiling of cancer progression using iTRAQ 3D LC mass spectrometry on high-quality serum samples to identify biomarkers of prostate cancer. Cystatin C was not found to be associated with prostate cancer progression in the study. A large-scale study initiated by Li et al. (54) explored the correlation between total prostate-specific antigen (PSA) and renal indicators such as cystatin C and creatinine in a Chinese ethnic minority, and showed a significant positive correlation between cystatin C levels and total PSA in the Mongolian population (p<0.0001). Thus, cystatin C may be used in combination with PSA to assist in the identification of prostate cancer in this ethnic group. A study by Zhao et al. (55) concluded that serum cystatin C could be a valid marker for the differential diagnosis of prostate cancer. Their model based on PSA, cystatin C, and neutrophil/lymphocyte ratio had excellent discriminatory performance (AUC = 0.913, sensitivity = 83%, specificity = 82%).

Perez-Cornago et al. (56) attempted to mine the UK Biobank (included more than 200,000 prostate cancer cases) for biomarkers associated with prostate cancer incidence and mortality. After limiting the follow-up duration, they found that cystatin C was the only biomarker that was negatively associated with the risk of prostate cancer. The authors suggested that this may be due to the fact that men with kidney disease have lower circulating testosterone concentrations, which indirectly reduces the risk of prostate cancer, an opinion that was consistent with Carrero et al. (57). Meanwhile, the authors did not find any biomarkers that were significantly associated with patient mortality. Fan et al. (46) evaluated data from 48 patients with castrate-resistant prostate cancer (CRPC) and found that when subjects receiving docetaxel-based chemotherapy had elevated pre-treatment serum cystatin C, their mortality was significantly higher. The high cystatin C group (>1.61 mg/l) had a median OS of 15.6 months, while the low cystatin C group had a median OS of 25.3 months (P < 0.001). The finding implied that serum cystatin C can be an independent indicator for feedback following docetaxel treatment and that it may be utilized to predict CRPC prognosis. Srour et al. (58) defined Growth Differentiation Factor-15, N-terminal pro-brain natriuretic peptide, glycated hemoglobin A1c, C-Reactive Protein, and cystatin C as aging-related markers. They attempted to find their relationship with the risk of cancer/cardiovascular disease development. One of these studies involved cystatin C and prostate cancer, but no statistical significance was found.

### Cystatin C expression in prostate cancer tissue

Jiborn et al. (59) analysed cystatin C level in radical prostatectomy specimens homogenated by Western blotting and enzyme-linked immunosorbent assay. The results showed that cystatin C levels were significantly higher in tumor tissues than in normal tissues, but the immunohistochemical expression of cystatin C in non-neuroendocrine prostate cancer cells gradually decreased with increasing Gleason grade. Meanwhile, cystatin C-positive neuroendocrine-like cells were stronger in prostate cancer than in benign tissues, suggesting a link between cystatin C and neuroendocrine differentiation in prostate cancer progression. Wegiel et al. (60) collected 448 specimens of benign and tumor tissues from prostate cancer patients who underwent radical prostatectomy, and determined the expression of cystatin C and its association with matrix metalloproteinases and androgen receptor using immunohistochemistry and tissue-microarray techniques. Almost all benign specimens showed significantly higher cytoplasmic protein expression of cystatin C, whereas cancerous tissues could barely be immunostained. The differences were statistically significant (P < 0.001), suggesting that cystatin C protein expression levels were generally downregulated in cancer tissues compared with the benign fraction of pathological specimens. Meanwhile, although not statistically significant, when assessing OS at 100 months, the authors still found that patients with low cystatin C expression in cancer tissue had a poorer prognosis than those with high levels (p = 0.307). In this study, it is proposed that targeted inhibition of cystatin C using specific siRNA resulted in an increased invasiveness of PC3 cells, whereas induction of cystatin C overexpression greatly reduced the invasion rate of PC3 *in vitro*. The effect of cystatin C on modulating the PC3 cell invasion was provoked by an ERK2 inhibitor that specifically inhibited MAPK/ERK2 activity.

### Urine cystatin C expression in prostate cancer

Guo et al. (61) identified 14 promising biomarkers linked to prostate cancer risk stratification and developed the 14-Gene Panel, a non-invasive tool. In two separate prospective and retrospective urine research cohorts, the scientists examined the biomarkers’ performance on tissue specimens and pre-biopsy urinary sediment. A quantitative real-time polymerase chain reaction was used to quantify the mRNA expression data of each biomarker in the urine sediment RNA samples of 202 patients. According to the 14 biomarkers studied, CST3 mRNA expression was considerably elevated in urine samples from higher-risk prostate cancer patients compared to those from the lower-risk group (P <0.0001).

## Cystatin C in bladder cancer

Fewer studies have examined the association between cystatin C and bladder cancer. The three articles we retrieved all focused on studying the link between serum cystatin C levels and the aggressiveness of bladder tumors. Because of the opposite conclusions in the only studies, there is still great research potential to study the association between cystatin C and bladder cancer.

Tokyol et al. (43) investigated the expression of cathepsin D in primary bladder cancer and attempted to establish its link with conventional pathological characteristics and serum cystatin C levels. The findings revealed that cathepsin D expression in tumor or stromal cells had no effect on serum cystatin C levels, and that their levels did not directly correlate with disease progression in primary bladder cancer. Between controls and patients, there were no significant differences in serum cystatin C levels (P > 0.05). Tokarzewicz et al. (44) employed a novel imaging technology named surface plasmon resonance imaging to detect 90 patients with bladder cancer and 27 healthy people. This method was used to measure the concentration of cystatin C in serum and urine. Serum levels of cystatin C from the patients were significantly lower than those in the controls (P < 0.001), whereas the cystatin C concentrations in urine were not significantly different from those of the controls. The findings suggest that serum cystatin C may be used as a potential biomarker for bladder cancer. We believe that the main limitation of this study is the difficulty in general clinical application of the new technique. Wang et al. (62) gathered clinical data from 425 bladder cancer patients’ records. Each group’s pre-treatment serum cystatin C levels were compared. Tumor parameters (tumor size, number of tumors, pathological features, all P>0.05) had no statistically significant changes in serum cystatin C levels. The authors found that circulating cystatin C is neither a reliable predictor of bladder cancer clinicopathologic characteristics nor a possible predictor of bladder cancer carcinogenesis. The result was consistent with Tokyol et al.

## Cystatin C and other urogenital malignancy

Mok et al. (63) quantified the association of eGFR (based on creatinine and cystatin C) and urinary albumin-to-creatinine ratio with the risk of cancer incidence using Cox regression models adjusted for potential confounders. Due to changes in prostate cancer guidelines during follow-up, the investigators excluded prostate cancer from parital analysis to reduce interference during cancer analysis. The study did not find any valuable links between eGFR based on cystatin C and the incidence risk of urologic neoplasms.

Stefanowicz et al. (64) examined single kidneys in 26 Wilms tumor patients (mean age, 11.17 years) clinically, biochemically, and sonographically. Single kidney function was assessed using cystatin C levels and compared with serum creatinine concentration and eGFR. Children with higher serum cystatin C concentrations had lower eGFR (P = 0.02) and lower parenchymal thickness/kidney length ratio (P = 0.0065), which might be because parenchymal thickness encompasses the portion of the kidney where the majority of the glomeruli are found. Renal hypertrophy was observed in 23 children and was associated with cystatin C level (P < 0.05). In addition, cystatin C levels may increase in subjects with normal GFR. As a result, cystatin C may aid in the early detection of children who need a more thorough evaluation of their renal function.

Since chronic kidney disease is a common complication resulting from chemotherapy (cisplatin-based) in patients with testicular cancer, Ichioka et al. (65) attempted to select markers more suitable for the assessment of renal function in patients with testicular cancer by comparing the ability of creatinine-based assessment of eGFR and cystatin C-based assessment of eGFR in the diagnosis of chronic kidney disease. The authors compared eGFR based on serum creatinine and cystatin C levels in 53 patients with testicular cancer and showed that creatinine-based eGFR was significantly lower than cystatin C-based eGFR (p<0.05). Thus, cystatin C-based assessment of GFR may overestimate renal function in TC survivors cured by cisplatin-based chemotherapy. Cameron et al. (66) found that patients with testicular cancer showed early signs of nephrotoxicity, such as elevated serum cystatin C, after 3 to 4 rounds of BEP (bleomycin, etoposide, cisplatin). However, the signs returned to baseline levels after three months with no significant impairment of long-term renal function.

Tan et al. (67) investigated the values of serum cystatin C in 538 patients with upper tract urothelial carcinoma following radical nephroureterectomy. For high and low cystatin C levels, the cut-off value was 1.4 mg/L. The findings revealed that individuals with preoperative higher cystatin C were older and had lower renal function than those with lower cystatin C (both P < 0.001). The group with elevated cystatin C had notably low survival outcomes (including OS, CSS, and recurrence-free survival). Elevated serum cystatin C was shown to be an independent risk predictor of OS (HR: 1.989, 95% CI: 1.366-2.896), CSS (HR: 1.997, 95% CI: 1.351-2.996), and recurrence-free survival (HR: 1.429, 95% CI: 1.009-2.023) in multivariate Cox analysis. In conclusion, patients with upper tract urothelial carcinoma who had an increased preoperative serum cystatin level had a considerably worse survival outcome. In 2021, Nishimura et al. (68) recruited 18 patients with advanced or metastatic urothelial carcinoma who were treated with a combination of gemcitabine and cisplatin. They used serum creatinine or serum cystatin C to compute eGFR and serum creatinine to estimate creatinine clearance. Based on urine and serum creatinine, the correlation, bias, accuracy, and creatinine height index between eGFR, or estimated creatinine clearance, and measured GFR based on creatinine clearance (mGFR) were computed. The serum cystatin C-based eGFR had the strongest correlation with mGFR, according to the findings. Furthermore, serum cystatin C-based eGFRs had considerably lower bias, mean error, mean absolute error, and root mean square error than serum creatinine and estimated creatinine clearance-based eGFRs. The relationship between serum cystatin C/mGFR-based eGFR and creatinine height index was less than the relationship between serum creatinine/mGFR-based eGFR and creatinine height index, suggesting that serum cystatin C is less affected by muscle mass. The authors conclude that serum cystatin C-based eGFR better represents renal function in uremic patients than serum creatinine-based eGFR, suggesting that serum cystatin C might be helpful in evaluating renal function of patients with advanced or metastatic urothelial carcinoma in clinical settings.

## Conclusion and perspectives

Cystatin C is a biomarker widely present in human serum, tissues, and urine. Numerous studies have shown its promising clinical application as a predictor of renal function and tumor prognosis. A large number of previous studies, including in the field of urogenital malignancy (**Table 1**), have shown that cystatin C is a valuable predictor of renal function, allowing better early prediction of renal impairment in patients with different cancers and may be more sensitive and accurate than creatinine (69–73). Multiple lines of evidence suggest that cystatin C is a promising biomarker in predicting early renal impairment after nephrectomy in patients with RCC. It can also assist renal function in patients with prostate cancer after radical prostatectomy. We also reviewed the prognostic correlation between cystatin C and urogenital malignancy. Although some studies have suggested that cystatin C levels in patients’ serum and tumor tissues, especially in RCC and prostate cancer, can be used as a potential marker of effectiveness evaluation after relevant treatment (**Table 2**) these findings are controversial due to small sample size. Some studies suggest that because serum cystatin C levels are significantly elevated in prostate cancer patients and highly expressed in prostate tumor tissues, its application could be valuable as an early discriminator between prostate cancer and benign prostate disease, but it is difficult to pry or even replace PSA in the early identification. Meanwhile, the pathogenesis of cystatin C in rare urogenital malignancies remains unclear. Considering the current situation, such as insufficient sample size and some conflicting conclusions, we believe that there is still room for progress in the research related to cystatin C in urogenital malignancy.

**Table 1:** Overview of studies in the relationship between pretreatment serum cystatin C level and renal function in patients with urogenital malignancy.

Number	Author	Type	Year	Cases	Stage	Treatment	Result	References
1	Duan et al.	RCC	2017	98	Non-metastatic	Partial Nephrectomy	The predictive value of CysC level at postoperative 6 hours for acute kidney injury was better than that of the intraoperative amount of negative fluid balance and average urine volume.	(47)
2	Zheng et al.	clear cell RCC	2020	50	T1N0M0-T2N0M0	Nephrectomy	Estimation of renal function by serum CysC was weakly correlated with rGFR, but the valuation was low.	(48)
3	Wenzel et al.	RCC	2021	195	Non-metastatic	Nephrectomy	Preoperative CysC levels can predict renal impairment after RCC surgery.	(40)
4	Südfeld et al.	PC	2016	179	–	Radical prostatectomy	In patients with normal preoperative renal function, administration of a median dose of 1000 ml of hydroxyethyl starch did not result in deterioration of the postoperative CysC-based-renal function.	(23)
5	Yordanova et al.	PC	2017	55	Metastatic	[^177^Lu]Lu-PSMA-617 radioligand therapy	CysC is one of the most reliable predictive markers of nephrotoxicity.	(45)
6	Yang et al.	PC	2017	173	All stage	–	CysC levels can predict renal function in prostate neoplasia patients but were not a biomarker for the development of prostate neoplasia and were not correlated with the clinicopathological characteristics of PC.	(24)
7	Stefanowicz et al.	Wilms tumor	2010	26	–	Unilateral nephrectomy	CysC levels may be increased in Wilms tumor patients with normal GFR.	(64)
8	Ichioka et al.	TC	2017	53	Metastatic	Chemotherapy (cisplatin-based)	eGFR based on CysC may overestimate the renal function in TC survivors cured by cisplatin-based chemotherapy.	(65)
9	Cameron et al.	TC	2020	53	–	Chemotherapy (cisplatin-based)	Elevated serum CysC levels may occur early after chemotherapy; they return to baseline levels after 3 months and do not affect long-term renal function.	(66)
10	Nishimura et al.	UC	2021	18	–	Chemotherapy (cisplatin-based)	eGFR based on CysC reflected renal function more accurately than eGFR based on Scr.	(68)

RCC, renal cell carcinoma; PC, prostate cancer; TC, testicular cancer; UC, urothelial carcinoma; CysC, cystatin C; Scr, serum creatinine; eGFR, estimated glomerular filtration rate; rGFR, radionuclide glomerular filtration rate.

**Table 2:** Overview of studies in pretreatment cystatin C levels and survival prognosis in patients with urogenital malignancy.

Number	Author	Type	Year	Cases	Stage	Treatment	Kind of sampling	Cut-off value	Cases of high-CysC group (%)	Multivariate Cox analysis	References
1	Guo et al.	RCC	2017	325	All stage	Nephrectomy	Serum	1.09	109 (34)	OS HR:1.59 (1.10-2.29), P= 0.012 PFS HR:3.50 (1.29-9.51), P=0.013	(49)
2	Zhao et al.	RCC	2020	354	All stage	Nephrectomy	Serum	1.105	36 (10)	OS HR:10.513 (2.539-43.522), P=0.001 CSS HR:4.944 (1.017-24.043), P=0.048	(41)
3	Bodnar et al.	RCC	2016	56	Metastatic	MMT (Everolimus)	Serum	1.15	44 (79)	OS HR:2.60 (1.03-2.60), P= 0.0428 PFS HR:2.85 (1.34-6.05), P=0.0065	(39)
4	Guo et al.	clear cell RCC	2018	253	All stage	Nephrectomy	tissues	29.65	210 (83)	OS HR:5.98 (0.8-44.33), P=0.079	(42)
5	Fan et al.	PC	2017	54	CRPC	Chemotherapy (Docetaxel+Prednisone)	Serum	1.61	23 (43)	OS HR:2.394 (1.135-3.757), P= 0.001	(46)
6	Tan et al.	UTUC	2019	538	No distant metastasis	RNU	Serum	1.4	162 (30)	OS HR:1.989 (1.366-2.896), P<0.001 RFS HR:1.429 (1.009-2.023), P=0.044 CSS HR:1.997 (1.331-2.996), P=0.001	(67)

RCC, renal cell carcinoma; PC, prostate cancer; UTUC, upper tract urothelial carcinoma; CRPC, castration-resistant prostate cancer; MMT, molecular-targeted therapy; RNU, radical nephroureterectomy; OS, overall survival; CSS, cancer-specific survival; PFS, progression-free survival; RFS, progression-free survival, HR, hazard ratio; CI, confidence interval.

## Author contributions

JW was responsible for the study design. LD and ZL participated in the collection and assembly of literature. LD and JW conducted the literature analysis and interpretation. The manuscript was drafted by LD. JW proofread the manuscript for important intellectual content. All authors contributed to the article and approved the submitted version.
